# Arginine methylation: the promise of a ‘silver bullet’ for brain tumours?

**DOI:** 10.1007/s00726-020-02937-x

**Published:** 2021-01-06

**Authors:** Sabrina F. Samuel, Antonia Barry, John Greenman, Pedro Beltran-Alvarez

**Affiliations:** Department of Biomedical Sciences, University of Hull, Hull, UK

**Keywords:** Arginine methylation, Brain tumours, Glioblastoma, Inhibitors, Post-translational modifications, Protein arginine methyltransferases

## Abstract

Despite intense research efforts, our pharmaceutical repertoire against high-grade brain tumours has not been able to increase patient survival for a decade and life expectancy remains at less than 16 months after diagnosis, on average. Inhibitors of protein arginine methyltransferases (PRMTs) have been developed and investigated over the past 15 years and have now entered oncology clinical trials, including for brain tumours. This review collates recent advances in the understanding of the role of PRMTs and arginine methylation in brain tumours. We provide an up-to-date literature review on the mechanisms for PRMT regulation. These include endogenous modulators such as alternative splicing, miRNA, post-translational modifications and PRMT–protein interactions, and synthetic inhibitors. We discuss the relevance of PRMTs in brain tumours with a particular focus on PRMT1, -2, -5 and -8. Finally, we include a future perspective where we discuss possible routes for further research on arginine methylation and on the use of PRMT inhibitors in the context of brain tumours.

## Introduction

Gliomas are the commonest malignancy of the central nervous system with the most devastating form of glioma being grade IV astrocytoma, known as glioblastoma (GBM) (Ostrom et al. 2018). Despite intense research efforts, the prognosis of GBM patients remains poor, with a median survival of 1.5 years following initial diagnosis. There has been minimal progress in increasing this life expectancy over the past decade. The only established risk factor for developing GBM is exposure to ionising radiation such as from previous cancer treatments (Ellor et al. 2014). Little is known about other risk factors associated with GBM, with only a small percentage of patients (< 5%) presenting with a germline predisposition, such as one associated with the neurofibromatosis type 1 syndrome (D'Angelo et al. 2019).

Treatment of GBM commonly includes radiation and chemotherapy, but recurrence is almost inevitable (The GLASS Consortium 2018). The first line of treatment for GBM is surgical removal, however, complete resection is often not possible due to the diffuse nature of the tumour and the inability to remove all traces of malignancy. Following resection, the patient receives adjuvant radiotherapy and, in many cases, the alkylating agent temozolomide (TMZ) (Stupp et al. 2009). This combination of treatment has demonstrated a 2-month increase in life expectancy from 12.2 to 14.6 months, and a 16% increase in 2-year survival (Stupp et al. 2009). Over the past decade, many drugs have entered clinical trials for treatment of gliomas including epidermal growth factor receptor (EGFR) targeted therapies (Rajaratnam et al. 2020), protein kinase B (Akt) inhibitors (Kaley et al. 2019; Wen et al. 2013), mutant isocitrate dehydrogenase (IDH1) inhibitors (Fan et al. 2020) and calcium channel inhibitors (Holdhoff et al. 2017). Despite intense research efforts, minimal changes to GBM patient life expectancy have been observed since the introduction of TMZ therapy over 10 years ago.

Very recently, arginine methylation (ArgMe) inhibitors have gained attention as possible, novel cancer therapies (Jarrold and Davies 2019) and some have entered clinical trials in the oncology setting (see section “Clinical trials involving PRMT inhibitors”). This review first introduces ArgMe and the enzymes responsible for this protein post-translational modification (PTM). Second, we provide an overview of the endogenous mechanisms for regulation of ArgMe and of synthetic ArgMe inhibitors, with a particular focus on recent developments. Finally, we discuss the relevance of ArgMe specifically in the setting of GBM and provide a future perspective.

## Arginine methylation and protein arginine methyltransferases

### Arginine methylation

Protein PTMs are key aspects of both epigenetics and signal transduction due to their ability to change cellular localisation, interactions and activity of proteins. Many types of modifications exist, including phosphorylation, SUMOylation, ubiquitination, acetylation and methylation, all of which have varied specificities and effects. Protein methylation is a PTM involved in a vast number of processes and a predicted 1% of the functional genome encodes for the enzymes catalysing protein methylation (Katz et al. 2003).

Methylation of proteins involves the transfer of a methyl group (CH_3_) onto either an arginine or lysine residue. Both types of post-translational methylation contribute to the exquisite control of gene expression through modification of histone proteins. Lys and Arg methylation also regulate a range of non-histone proteins including proteins involved in transcription and RNA-binding, translation, chaperone, cytoskeletal and membrane proteins and adaptor/scaffold proteins (Guo et al. 2014; Lim et al. 2020). However, there are several and important differences between the methylation of lysines and arginines. First, Lys methylation is highly dynamic and there are eight families of lysine demethyltransferases (Jones et al. 2019) whereas methylation of Arg is generally considered a stable mark (Bedford and Clarke 2009). Second, Lys residues can be mono-, di- and tri-methylated while Arg residues can only be mono- and di-methylated (see below), this is important, because the different methyllysine and methylarginine isomers can have very different impacts at the molecular level. Lastly, methylation of Lys is less common than methylation of Arg on the proteomic scale (Guo et al. 2014; Hornbeck et al. 2015). The scope of the present review is to define the relevance and possible regulation of arginine, but not lysine, methylation in brain tumours.

ArgMe was first identified in 1967 (Paik and Kim 1967), but its significance has only been fully acknowledged in recent years (Blanc and Richard 2017). The availability of research tools including antibodies and specific inhibitors has allowed advances in the basic understanding of ArgMe as well as in its role in health and disease. The side chain of arginine comprises an aliphatic chain of three carbon atoms, ending with a guanidino group. This structure allows arginine to form π-stacking interactions with aromatic amino acids and nucleic acids (Gallivan and Dougherty 1999). Arginine has the highest p*K*_a_ value (13.8) of all amino acids and as such is positively charged at physiological pH (Fitch et al. 2015). The addition of a methyl group removes a potential hydrogen bond donor from the recipient arginine and produces a bulkier and more hydrophobic residue (Bedford and Richard 2005). Although the overall cationic charge is maintained, it is dispersed towards the added methyl groups, increasing affinity of arginine for three-dimensional aromatic cages consisting of clusters of aromatic amino acids (Tripsianes et al. 2011; Beaver and Waters 2016). This can either improve or hinder interactions with other proteins or nucleic acids.

There are a number of proteins responsible for ArgMe recognition (Guccione and Richard 2019) and the most studied contain so-called Tudor domains, with approximately 30 of Tudor-domain containing proteins encoded in the human genome (Côté and Richard 2005; Chen et al. 2011). Tudor domains contain numerous aromatic amino acids including tryptophan and phenylalanine, which form interactions with the guanidino group of methylarginine, but not with the guanidino group of unmethylated arginine (Tripsianes et al. 2011). Hydrogen bonding between the two entities also facilitates the interaction. Proteins that contain Tudor domains include Survival of motor neuron (SMN), Splicing factor 30 (SPF30), and Tudor domain-containing proteins (TDRD1/2/3/6/9 and 11). The proteins responsible for ArgMe recognition act as the *effectors*, or *readers* of ArgMe, and effectively translate specific methylation marks into defined molecular events such as gene transcription, mRNA splicing and a number of biochemical pathways (Yang and Bedford 2013).

### Protein arginine methyltransferases

**Fig. 1:**
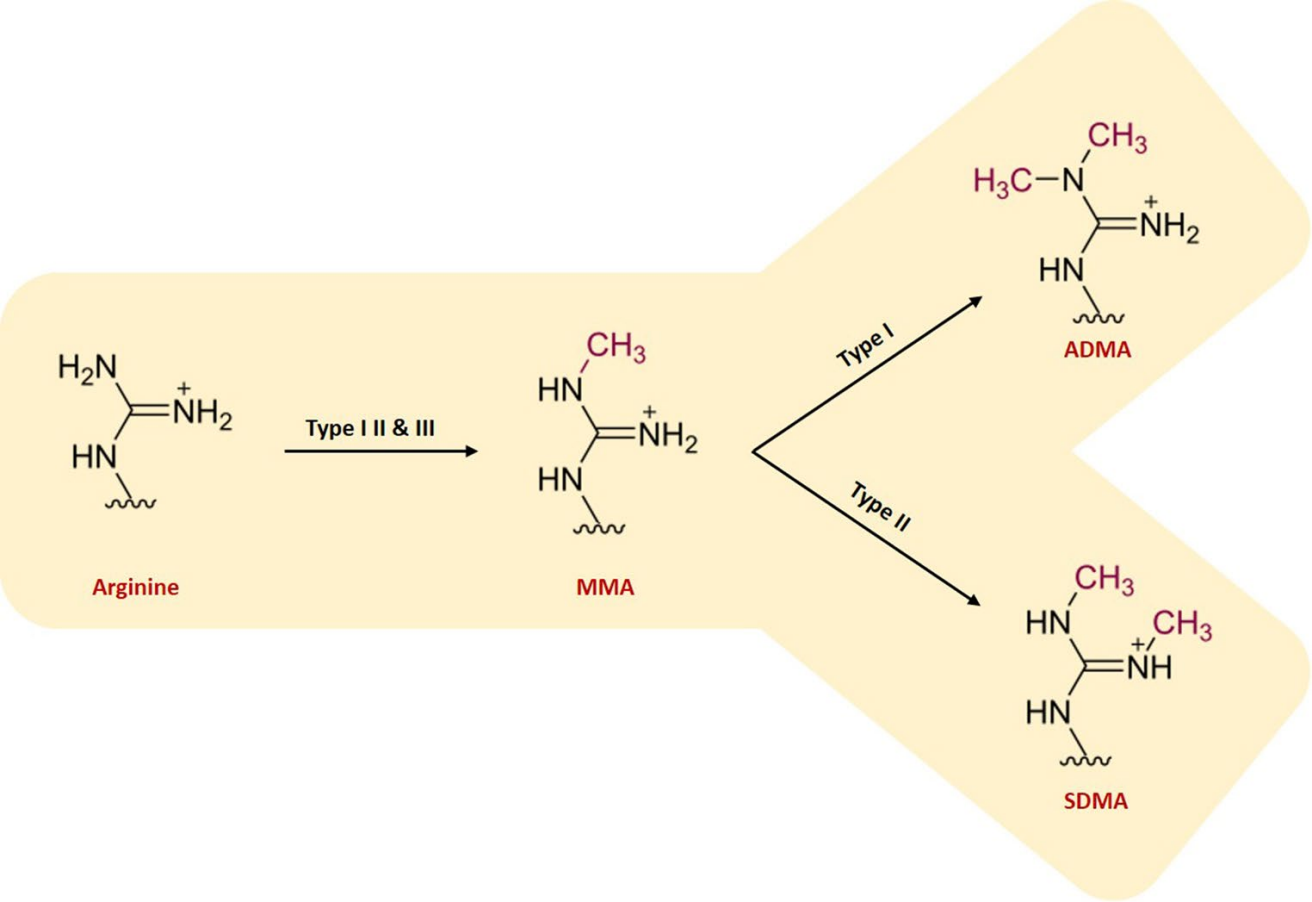
Scheme showing the different enzymatic activities of type I, II and III PRMTs. Type I, II and III PRMT enzymes catalyse the transfer of a methyl group from SAM to the side chain nitrogen on arginine residues on the target protein to produce monomethylarginine (MMA). Type I enzymes then catalyse the transfer of a further methyl group, asymmetrically onto the same nitrogen atom that results in asymmetric dimethylarginine (ADMA). Type II enzymes catalyse the transfer of a second methyl group symmetrically, onto the opposite nitrogen on the side chain of arginine (symmetric dimethylarginine, SDMA)

Protein arginine methyltransferases (PRMTs) are the enzymes responsible for the methylation of arginine residues (Yang and Bedford 2013). PRMTs catalyse the transfer of a methyl group from *S*-adenosyl-l-methionine (SAM/AdoMet) onto the side chain nitrogen of arginine, producing the by-product *S*-adenosyl-l-homocysteine (SAH/AdoHcy). There are three types of PRMTs that catalyse this reaction, each responsible for a different ArgMe end-product: Type I PRMTs lead to asymmetric dimethylarginine (ADMA); Type II PRMTs produce symmetric dimethylarginine (SDMA); and Type III PRMTs form monomethyl arginine (MMA) only (Fig. 1). As such, all three types of PRMTs transfer a single methyl group onto the target arginine, and only types I and II transfer a second one. Type I PRMTs include PRMT1, -2, -3, -4, -6 and -8. PRMT5 and -9 are type II PRMTs. PRMT7 was initially described as a Type II PRMT and subsequently characterised as type III (Zurita-Lopez et al. 2012) but recent reports have again reported Type II activity (Jeong et al. 2020; Vuong et al. 2020; Liu et al. 2020a).

Most PRMTs are ubiquitously expressed both at the cellular compartmentalization, and tissue expression levels. The most notable exception is PRMT8, which is expressed in the central nervous system (CNS) only (Lee et al. 2005). PRMT8 is also almost exclusively localised to the plasma membrane and this specific membrane expression is determined by a combination of N-terminal basic residues, post-translational modification by myristoylation (also at the N terminus), and PRMT8 dimerisation (Park et al. 2019). PRMT6 localises mainly to the cellular nuclei (Frankel et al. 2002). Most other PRMTs have been found both in nuclear and cytosolic fractions and their distribution often depends on the particular PRMT splice variant, PRMT–protein interactions and, of note, disease processes such as cancer (Wang et al. 2019a, b). This underscores the importance to achieve a fine regulation of PRMT activity. In the sections below, we discuss recent advancements in the understanding of how PRMTs are regulated by endogenous mechanisms and can be modulated by synthetic inhibitors, and the relevance of specific PRMTs in the context of gliomas.

## Regulation of arginine methylation

### Alternative splicing

Many PRMTs undergo alternative splicing during mRNA maturation, resulting in differences in the protein primary sequence. This can have consequences in terms of enzyme function and localisation, as has been reported for an isoform of PRMT1, named PRMT1v2. This isoform is uniquely localised to the cytoplasm, due to the presence of a functional nuclear export sequence (Goulet et al. 2007). PRMT2 also forms multiple isoforms following alternate splicing, producing PRMT2α, PRMT2β, PRMT2γ, PRMT2L2, as well as the full-length PRMT2 protein. The alternative PRMT2 isoforms have reduced methyltransferase activity compared with the full-length molecule, and varied localisation due to losses within domain III and the THW loop (Zhong et al. 2012). Two isoforms of coactivator-associated arginine methyltransferase (CARM1), also known as PRMT4, have been identified in human tissue: full-length CARM1 and a shorter CARM1Δ15 isoform. Exon 15 of CARM1 is excluded in this shorter isoform, resulting in the loss of auto-methylation capacity, but no change in methyltransferase activity (Wang et al. 2013).

### Micro-RNAs

Micro-RNAs (miRNA) are short non-coding lengths of RNA that function by regulating gene expression via base-pairing with complementary mRNA sequences (Jin et al. 2019). PRMTs can be regulated by miRNAs by binding to 3′ untranscribed regions (3′-UTRs) of PRMTs. For example, miR-4518, miR-92, miR-96, miR-32 and miR-19, all target PRMT5 mRNA and prevent its translation, ultimately leading to reduced cell proliferation (Pal et al. 2007; Wang et al. 2008; Lu et al. 2018). MiR-543 has been shown to bind to the 3′-UTR of PRMT9, which inhibits PRMT9 expression and leads to reduced PRMT9-driven cell oxidative phosphorylation and increased hypoxia-induced factor-1α (HIF-1α) stability in osteosarcoma cells (Zhang et al. 2017). PRMT1 translation is inhibited by binding of miR-503 and this is linked with a reduced epithelial–mesenchymal transition in hepatocellular carcinoma cells (Li et al. 2015). Similarly, MiRNA-195 reduces CARM1 expression, which is associated with decreased proliferation in colorectal cancer cells (Zheng et al. 2017). Of note, PRMTs have recently been shown to play an important role in the regulation of miRNA synthesis (Spadotto et al. 2020), which could open a new avenue for self-regulation of PRMT expression.

### Post-translational modifications of PRMTs

The effect of several types of PTMs, including phosphorylation, ubiquitination and methylation, on PRMT activity and stability have been documented. Phosphorylation of PRMTs can enable, inhibit or switch their methyltransferase activity, depending on the site of modification. For example, phosphorylation of PRMT5 at T132, T139 and T144 is required for its activity, whereas phosphorylation of Y304 and Y307 downregulates methyltransferase activity by disrupting the PRMT5 interaction with the WD-repeat methylosome protein MEP50 (Liu et al. 2011). Phosphorylation of PRMT5 at residue S15 by protein kinase C is induced by interleukin-1β and is required for the PRMT5 mediated activation of nuclear factor kappa-light-chain-enhancer of activated B cells (NF-κB) (Hartley et al. 2020), a major transcription factor involved in the innate and adaptive immune response (Lawrence 2009).

PRMT1 and CARM1 are also phosphorylated. Casein kinase 1 isoform alpha 1 phosphorylates PRMT1 between the regions 55–57, 102–105 and 284–289 to control PRMT1 targeting to chromatin and, therefore, regulate self-renewal pathways by changing gene expression (Bao et al. 2017). PRMT1 is also phosphorylated at Y291, which alters its substrate specificity (Rust et al. 2014). CARM1 is phosphorylated at residues S217 and S229. Phosphorylation inhibits methyltransferase activity in different ways, either through promoting cytoplasmic localisation (Feng et al. 2009) or preventing dimerization (Higashimoto et al. 2007), respectively. CARM1 is also phosphorylated at S572 by p38γ mitogen-activated protein kinase (MAPK). This inhibits CARM1 translocation to the nucleus, in turn inhibiting paired box protein 7 (PAX7) methylation, activation of myogenic factor 5 and subsequent induction of myogenesis (Chang et al. 2018).

Like many other proteins, PRMTs are also subject to degradation by the proteasome. PRMT1, CARM1 and PRMT5 for example, are substrates of ubiquitin E3 ligases. PRMT1 is ubiquitinated by E4B (Bhuripanyo et al. 2018), CARM1 by Skp, Cullin, F-box-containing complex (SCF) (Shin et al. 2016), and PRMT5 by carboxy-terminus of Hsc70-interacting protein (CHIP) ligase (Zhang et al. 2016). Recently, an orphan F-box protein, FBXO24, has been shown to modify PRMT6 by ubiquitination at K369 (Chen et al. 2020). Although questions remain about the specific ubiquitination sites on other PRMTs, it is generally accepted that modification by (poly)ubiquitination leads to proteasomal degradation of PRMTs (Hartley and Lu 2020).

Some PRMTs self-methylate to control their function. These include CARM1, PRMT6 and PRMT8. CARM1 is automethylated at R551 within its C-terminal domain. This modification does not alter enzyme activity, but facilitates protein–protein interactions required for transcriptional regulation of other proteins (Kuhn et al. 2011). PRMT6 can also undergo automethylation, specifically at R35. This modification is required for PRMT6 stability and, in the context of disease, for the inhibition of human immunodeficiency virus-1 (HIV-1) replication (Singhroy et al. 2013). PRMT8 is automethylated within its N-terminal domain (Sayegh et al. 2007), which results in blockage of the catalytic site and inhibition of further methylation (Dillon et al. 2013). Asymmetrical methylation of PRMT5 by CARM1 at R505 increases PRMT5 oligomerisation and is critical for PRMT5 methyltransferase activity (Nie et al. 2018).

Glutathionylation is a PTM that can target proteins exposed to oxidative stress and can also modulate protein structure and function. Cys sulfhydryl groups are particularly responsive to the redox state of cells and can be post-translationally modified by glutathionylation. Recently, glutathionylation at C42 has been reported to decrease methyltransferase activity of PRMT5 by affecting PRMT5-MEP50 interactions (Yi et al. 2020). PRMT5 C42 glutathionylation was increased in aged mice and in cell lines treated with H_2_O_2_ and the modification was reversed by Glutaredoxin-1. This finding contributes to growing evidence that PRMT activity can be affected by oxidative stress (Morales et al. 2015).

### Cross-talk between ArgMe and other PTMs

Over the past few years, the interplay between ArgMe and other PTMs has become increasingly clear. This interplay, or cross-talk, is a hallmark of the histone code in epigenetic regulation of gene expression (Guccione and Richard 2019). In non-histone proteins, the PTM most commonly shown to cross-talk with ArgMe is phosphorylation. For example, PRMT5-mediated methylation of apoptosis signal-regulating kinase 1 (AKS1) at R89 interacts with S83 phosphorylation by Akt (Chen et al. 2016). Cross-talk between ArgMe and phosphorylation has also been described in voltage-gated ion channels (Beltran-Alvarez et al. 2015; Onwuli and Beltran-Alvarez 2016). The possible role of cross-talk between ArgMe and phosphorylation in cancer has been an outstanding question in the field since the publication of the classic paper by Hsu and coworkers that described cross-talk between EGFR R1175 methylation and Y1173 phosphorylation, which was reported to modulate cell proliferation, migration and invasion of EGFR-expressing cells (Hsu et al. 2011). Recently, the relevance of ArgMe-phosphorylation cross-talk in regulation of cancer stem cell properties has been investigated in the context of Lymphoid-specific helicase (LSH). Mimicking constitutive phosphorylation of LSH at S503 (by a S503D mutant) downplayed LSH ArgMe at R309, and this was associated with the activation of stem cell-like gene expression in PC-9 lung cancer cell lines (Liu et al. 2020b).

There is also emerging interest in the cross-talk between different types of ArgMe, mainly between Type I and Type II PRMT activity. This springs from the observations that, first, inhibitors targeting both types of PRMT have synergistic effects (Fedoriw et al. 2019; Fong et al. 2019; Gao et al. 2019), and second, that inhibiting or knocking down specific PRMTs leads to increases in other ArgMe events, catalysed by other PRMTs (Musiani et al. 2019; Hartel et al. 2019). In summary, cross-talks between ArgMe and other PTMs entail a different form of regulation from those discussed elsewhere in this section and put the emphasis on the substrate rather than being PRMT-centred. We predict that the importance of cross-talk will gain visibility because of the large number of residues in the vicinity of known ArgMe sites that are post-translationally modified by e.g. phosphorylation, ubiquitination, acetylation and lysine and arginine methylation (Onwuli et al. 2017).

### PRMT–protein interactions

PRMT5 is a member of a multimeric complex and interacts with many cofactors that regulate its activity. The most important cofactor of PRMT5 is methylosome protein 50 (MEP50), with which it creates a hetero-octameric structure. The PRMT5:MEP50 complex binds further cofactors including swelling-induced chloride conductance regulatory protein (plCln) and serine/threonine kinase RioK1, allowing for the recruitment of distinct methylation substrates (Guderian et al. 2011). Recently, oxidation resistance gene 1 (OXR1A) has been identified as an activator of PRMT5 activity, at least in vitro (Yang et al. 2020).

Other PRMT–protein interactions contribute to the regulation and substrate specificity of PRMTs. These include human CCR4-associated factor 1 (hCAF1), which inhibits PRMT1 methylation of Src-associated substrate in Mitosis of 68 kDa (Sam68) and H4, but not hnRNP1 (Robin-Lespinasse et al. 2007). hCAF1 is found within the carbon catabolite repression—negative on TATA-less (CCR4-NOT) complex, suggesting that PRMT1:hCAF1 interactions have a role in the crosstalk between transcriptional regulation and RNA metabolism (Morales et al. 2016). Another example is actin filament-associated cytoskeletal regulatory protein differentially expressed in adenocarcinoma of the lung (DAL-1/4.1B), which is known to inhibit PRMT3 (Singh et al. 2004) but allows substrate-specific PRMT5 methylation of myelin basic proteins (Jiang et al. 2005). Other PRMT:protein interactions can facilitate PRMT recognition of substrates, for instance nucleosomal methylation activator complex (NUMAC), which targets CARM1 to H3 in vivo (Xu et al. 2004). Other examples of PRMT protein partners are high mobility group AT-Hook 1 (HMGA1) and CCCTC-binding factor (CTCFL), which increase the methylation activities of PRMT6 (Lo Sardo et al. 2013) and PRMT7 (Jelinic et al. 2006), respectively. PRMTs have also been found forming homomers and heteromers with other PRMT isoforms, generally to enhance PRMT activity (Pak et al. 2011).

### Possible arginine demethylases

Lysine demethylation is now a well-established activity and numerous responsible enzymes have been identified, of which there are two families: flavin-dependant methyllysine demethylases and Fe(II) and 2-oxoglutarate-dependant Jumonji C (JmjC)-domain-containing enzymes (Böttger et al. 2015). As of yet, however, no arginine demethylation enzymes have been confirmed. JMJD6 was thought to be an arginine demethylase, which acted on histone H3R2 and H4R3 (Chang et al. 2007). However, subsequent studies showed that JMJD6 was in fact a hydroxylase that acts predominantly on nucleic acids (Hong et al. 2010). The exact activity and role of JMJD6 is controversial and remains unclear (Böttger et al. 2015), although it has been shown that JMJD6 can demethylate the stress granule nucleating protein G2BP1 in vivo (Tsai et al. 2017). Particular lysine demethylases (KDM4E, KDM4A, KDM5C and JMJD1B) have also been shown to also have arginine demethylase activity in vitro (Walport et al. 2016; Li et al. 2018).

The degradation of proteins bearing methylated arginine residues leads to the proteolytic products N^G^-monomethyl-l-arginine (monomethylarginine, mMA), N^G^,N^G^-dimethyl-l-arginine (asymmetric dimethylarginine, aDMA) and N^G^,N′^G^-dimethyl-l-arginine (symmetric dimethylarginine, sDMA) (Tsikas et al. 2018). These metabolites exercise a range of functions when released into circulation, notably related to nitric oxide synthase inhibition. sDMA is primarily eliminated through renal excretion while mMA and aDMA are mainly excreted as mono- and di-methylamine, respectively (Said et al. 2019). The enzyme responsible for the hydrolysis of mMA and aDMA into mono- and di-methylamine, and citrulline, is dimethylarginine dimethylaminohydrolase (DDAH) (Jarzebska et al. 2019). The metabolism of aDMA and sDMA can also include transamination into asymmetric or symmetrical α-keto-dimethylguanidinovaleric acid, catalysed by alanine:glyoxylate aminotransferase 2 (AGXT2), (Jarzebska et al. 2019). However, DDAH and AGXT2 activities have only been observed towards methylarginine metabolites, and not towards proteins modified by ArgMe and, therefore, DDAH and AGXT2 cannot be considered true protein arginine demethylases. Similarly, peptidyl-arginine deaminases (PADs) are enzymes that convert methylarginine into citrulline through hydrolysis (Wang et al. 2004) but citrulline has different chemical properties to unmethylated arginine and PADs cannot, therefore, be considered authentic demethylases either.

### PRMT inhibitors

Allantodapsone and stilbamidine are PRMT1 inhibitors found through the virtual screening of 1990 compounds using a homology model of human PRMT1 and *Aspergillus nidulans* RmtA (fungal PRMT homolog) created using the rat PRMT3 X-ray structure (Spannhoff et al. 2007a). Thirty-six compounds shown to have suitable docking into the binding pocket of the structure were tested in an in vitro assay to evaluate their ability to inhibit RmtA. Allantodapsone was found to inhibit PRMT1 with an IC50 of 1.7 µM. Compounds discovered in this study share a basic motif that mimics the guanidine nitrogen of the substrate protein. Other PRMT1 inhibitors have also been identified using similar approaches (Bissinger et al 2011), including RM65 (Spannhoff et al. 2007b) and A9 (Wang et al. 2012a).

Due to the similarities between the guanidine structure in substrate arginines and the amidine group of diamidine-based compounds, as well as the existence of the diamidine PRMT inhibitor stilbamidine, Yan et al. explored the use of such compounds as PRMT inhibiting drugs (Yan et al. 2014b). Furamidine (also known as DB75) was identified to selectively inhibit PRMT1, with an IC50 of less than 10 µM for PRMT1 and higher than 160 µM for PRMT5—and even greater for CARM1 and PRMT6. Furamidine is thought to act as a competitive substrate inhibitor (Yan et al. 2014b).

The compounds EPZ020411 and CMPD-1 (inhibitors of PRMT6 and CARM1, respectively) contain an ethylenediamine moiety which is thought to mimic the structure of arginine. Experiments with negative control compounds lacking this ethylenediamine group confirmed the importance of this moiety through a lack of PRMT inhibition. The cyclobutoxy group found in EPZ020411 was thought to provide the specificity against PRMT6. Following this hypothesis, its replacement with a smaller functional group (i.e. isopropoxy) allowed to broaden the inhibitory of EPZ020411 actions against other type I PRMTs. This approach was used to rationally design a broader PRMT type I inhibitor, MS023. MS023 was found to have a strong inhibitory effect against a plethora of type I PRMTs exclusively with IC50 in the nM range; including PRMT1 (30 nM), PRMT3 (119 nM), CARM1 (83 nM), PRMT6 (4 nM) and PRMT8 (5 nM), (Eram et al. 2016).

Type II PRMT inhibitors have also been developed and extensively investigated. In a landmark paper by a multi-pharmaceutical collaboration, a fluorescence assay was used to monitor the monomethylation of the PRMT5 target H4R3 and to screen a library of small molecules. Following re-testing and counter screens, a subset of 17 compounds was identified with IC_50_ ranging between 0.4 and 7 µM (Chan-Penebre et al. 2015). The most successful compound was EPZ007345 which was then structurally developed to increase potency and other pharmacokinetically favourable characteristics including absorption, distribution, metabolism and excretion. The end product, EPZ015666 (GSK3235025), acts as a competitive substrate inhibitor of PRMT5 with an IC_50_ of 22 nM in vitro and 64–902 nM in cell assays. A second study optimised the structure for use as an in vitro tool leading to EPZ015866, which has an IC_50_ of 4 nM for PRMT5 (Duncan et al. 2015).

In contrast to these substrate-binding inhibitors, LLY-283 is a SAM pocket competitive inhibitor of PRMT5 with an IC50 of 22 nM which acts by inhibiting the formation of the PRMT5:MEP50 complex (Bonday et al. 2018). When tested in vivo, the drug had an IC_50_ of 25 nM, measured by the levels of methylated SmBB. Recently, a novel allosteric inhibitor of PRMT5 has been reported, which leads to occlusion of both the SAM and substrate-binding sites through displacement of the loop ELLGSFADNEL spanning PRMT5 residues 435–445 (Palte et al. 2020). The question of whether the combination of Type I and Type II inhibitors would synergise against tumour cells is obvious and has recently been addressed by several research groups (Fedoriw et al. 2019; Fong et al. 2019; Gao et al. 2019). Both groups have independently reported that the combination of PRMT1 and PRMT5 inhibition has synergistic effects on tumour cell growth, at least partly mediated by methylthioadenosine phosphorylase (MTAP).

PRMT7, the only recognised Type III PRMT, has also been targeted for development of specific inhibitors. DS-437 is a SAM analogue inhibitor of PRMT5 that also shows activity against PRMT7 (Smil et al. 2015). More recently, specific PRMT7 inhibition by SGC8158, a SAM-competitive inhibitor, has been described (Szewczyk et al. 2020). A list of inhibitors sorted by their PRMT targets is shown in Table 1.

**Table 1:** Summary of preclinical PRMT inhibitors

PRMT	Inhibitors	Target site
PRMT1	Furamidine	Competitive substrate inhibitor
	MS023	Type I competitive substrate inhibitor
	Allantodapsone	Competitive substrate inhibitor
	Stilbamidine	Competitive substrate inhibitor
	RM65	Competitive substrate inhibitor
	A9	Competitive substrate inhibitor
PRMT2	MS023	Type I competitive substrate inhibitor
PRMT3	MS023	Type I competitive substrate inhibitor
PRMT4/CARM1	MS023	Type I competitive substrate inhibitor
	CMPD-1	Competitive substrate inhibitor
PRMT5	JNJ-64619178	Small molecule inhibitor of SAM and substrate binding pockets
	LLY-283	SAM competitive inhibitor
	EPZ015866/GSK-591	Competitive substrate inhibitor
	DS-437	SAM analogue inhibitor
PRMT6	MS023	Type I competitive substrate inhibitor
	EPZ020411	Competitive substrate inhibitor
PRMT7	SGC8158	SAM competitive inhibitor
	DS-437	SAM analogue inhibitor
PRMT8	MS023	Type I Competitive substrate inhibitor

### Clinical trials involving PRMT inhibitors

**Fig. 2:**
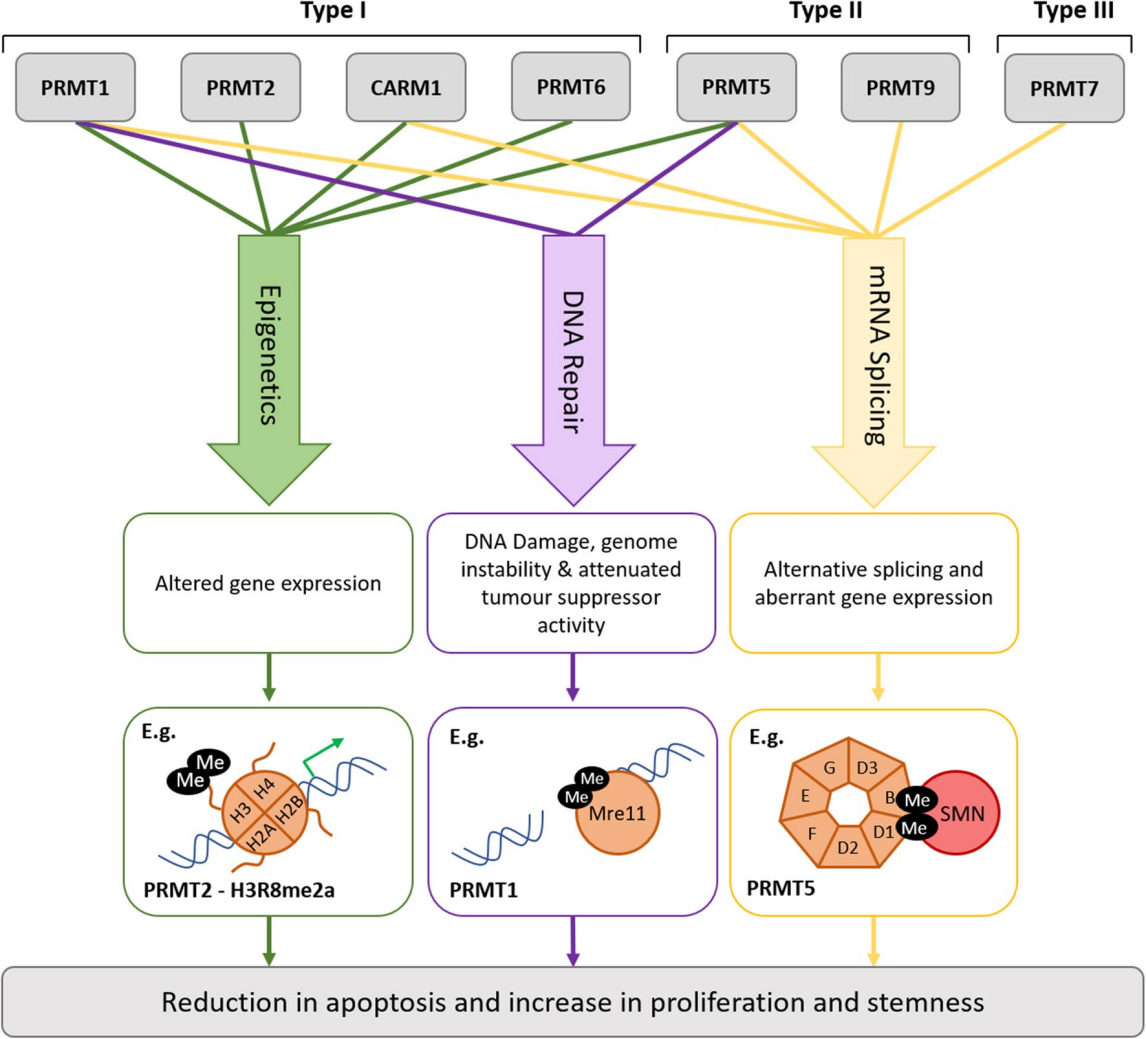
Schematic of the mechanisms by which ArgMe leads to oncogenesis. PRMTs and their alternatively spliced isoforms have diverse roles in transcriptional regulation, splicing and DNA damage repair, through MMA, SDMA and ADMA on specific targets. PRMTs methylate a combination of histone and non-histone targets. For example, PRMT2 methylates H3R8, inducing gene expression. PRMT1 methylates double strand break repair protein meiotic recombination 11 homolog (Mre11), anchoring it to the double strand break. PRMT5 methylates Sm ribonucleosomal proteins, promoting uridine-rich small nuclear ribonuclear protein (UsnRNPs) and survival motor neuron (SMN) spliceosomal complex assembly. Methylation of these targets results in the increased expression of oncogenic genes and the attenuation of tumour suppressive genes, either through promoter activation or repression by epigenetic regulation, alterations in splicing patterns or through an increase in genomic instability. PRMT activity has also been shown to promote tumour stem cell characteristics

As highlighted in the sections above and summarised in Fig. 2, PRMTs play key roles in many cellular processes and PRMT dysregulation has been associated with cancer, which has prompted the development of PRMT inhibitors into Phase I clinical trials. These clinical trials involve several tumour types, including a range of blood and solid cancers, and are summarised in Table 2. Over the past few years, there has been great interest in understanding the mechanistic basis that supports the use of PRMT inhibitors in cancer. As described above (see “Arginine methylation” section), a prominent role of PRMTs is as RNA-binding proteins and in RNA splicing. In cancer, RNA splicing is often dysregulated including by mutations in RNA splicing factors (RNA-SF), and inhibition of Type I PRMT and PRMT5 by MS023 and GSK591, respectively, has been shown to effectively target cells bearing RNA-SF mutations, both in vitro and in vivo (Fong et al. 2019). GSK3368715, an inhibitor of Type I PRMTs, alters exon utilisation and RNA splicing most likely by inhibiting ArgMe of heterogeneous nuclear ribonuclear (hnRNP) proteins (Fedoriw et al. 2019). The PRMT5 inhibitor GSK3326595 has been shown to promote the alternative splicing of the human ortholog of mouse double minute 4 (MDM4), which leads to the activation of the tumour suppressor p53 protein and reduced tumour cell viability (Gerhart et al. 2018). GSK3326595 was developed from GSK3235025 (Chan-Penebre et al. 2015) with both compounds having potent anti-proliferative effects both in vitro and in vivo (Chiang et al. 2017; Gerhart et al. 2018). These basic and translational science efforts have been paralleled by much interest from large pharmaceutical companies in developing and trialling PRMT inhibitors, including in gliomas and GBM populations as expansion cohorts (Table 2).

**Table 2:** Current clinical trials taking place involving PRMT inhibitors (from clinicaltrials.gov, November 2020)

Compound name	Target PRMT	Dose escalation cohort	Expansion cohort	Trial identifier
GSK3368715	Type I PRMTs except PRMT3	Relapsed/refractory diffuse large B-cell lymphoma and selected solid tumours with frequent MTAP deficiency	Diffuse large B-cell lymphoma and relapsed/refractory solid tumours including pancreatic, bladder, and non-small cell lung cancer	NCT03666988
EPZ015938 (GSK3326595)	PRMT5	Non-Hodgkin’s lymphoma and solid tumours	Triple-negative breast cancer, metastatic transitional cell carcinoma, recurrent GBM, non-Hodgkin’s lymphoma p53 mutant gene, adenoid cystic carcinoma, hormone receptor-positive adenocarcinoma of the breast, human papillomavirus positive solid tumours of any histology (including cervical cancer and squamous cell carcinoma of the head and neck) and P53 wild-type non small-cell lung cancer	NCT02783300
EPZ015938 (GSK3326595)	PRMT5	Myelodysplastic syndrome and acute myeloid leukaemia	Newly diagnosed myelodysplastic syndrome	NCT03614728
JNJ-64619178	PRMT5	Non-Hodgkin’s lymphoma and solid tumours	Myelodysplastic syndromes	NCT03573310
PF-06939999	PRMT5	Advanced solid tumours (non-small cell lung cancer, head and neck squamous cell carcinoma, oesophageal cancer, endometrial cancer, cervical cancer, and bladder cancer)	Advanced solid tumours	NCT03854227
PRT811	PRMT5	Advanced cancers and high-grade gliomas	Advanced solid tumours and GBM	NCT04089449

## Arginine methylation in GBM

### PRMT1 in GBM

PRMT1 is the predominant protein arginine methyltransferase responsible for asymmetric methylation and was first discovered as a binding partner to nerve growth factor (NGF)-inducible protein TIS21 and B-cell translocation gene BTG1 (Lin et al. 1996; Tang et al. 2000). Partial loss of the protein in mice embryonic fibroblasts results in loss of proliferation and a drastic increase in genomic instability. Complete loss of PRMT1 is embryonically lethal in mice, suggesting a multifunctional capacity within the cell (Pawlak et al. 2000). PRMT1 is predominantly localised to the nucleus but also has substrates found in the cytoplasm and other cellular compartments (Herrmann et al. 2005), and has in fact been shown to play a role in protein shuttling between these areas (Herrmann et al. 2004).

PRMT1 has been shown to take part in cell signalling through the Akt pathway. The growth factor signalling receptor EGFR is responsible for activation of the Akt pathway and has been found to be methylated by PRMT1 (Wang et al. 2019a, b). EGFR methylation at R198 and R200 augments ligand binding and enhances receptor activation. PRMT1 also methylates estrogen receptor-alpha (ERα) at R260, enabling the formation of the ERα/S-locus cysteine-rich proto-oncogene tyrosine-protein kinase (Scr)/phosphoinositide-3-kinase (PI3K) complex (Le Romancer et al. 2008). TGFβ signalling is also influenced by PRMT1 activity. PRMT1 methylates the inhibitory protein small worm phenotype mothers against decapentaplegic 6 (SMAD6) at R74, allowing the recruitment of the Bone Morphogenetic Protein (BMP) effectors SMAD1 and SMAD5 (Xu et al. 2013). PRMT1 also methylates the inhibitory proteins SMAD6 and SMAD7 (at R57 and R67), leading to activation of SMAD3 (Katsuno et al. 2018). These activities have a crucial role in epithelial–mesenchymal transition and epithelial stem-cell generation.

PRMT1 expression is considerably high in the foetal brain, however, it is reduced post-maturation of the adult brain, which suggests a role in development (Huang et al. 2011; Pawlak et al. 2000; Ikenaka et al. 2006). Research has proposed a role for PRMT1 in astrocytic differentiation through the methylation of signal transducer and activator of transcription 3 (STAT3) (Honda et al. 2017). PRMT1 is upregulated in both GBM tissue and cell lines, including U-87MG, U-251 and A172, at both the RNA and protein level (Wang et al. 2012c). Its RNA expression has been correlated with poor patient survival (Dong et al. 2018). PRMT1 knock-down through siRNA causes a loss of cell proliferation as observed through a reduction of S-phase cells by flow cytometry and also by MTT assay. Induction of apoptosis was also observed by TUNEL assay (Wang et al. 2012c). More recently, inhibition of PRMT1 by furamidine has been suggested to reduce GBM cell viability (Samuel et al. 2018).

PRMT1 is recruited by chromatin-associated proteins to induce the expression of proliferative genes (Takai et al. 2014). Chromatin target of PRMT1 (CHTOP), when associated with 5-hydroxymethylcytosine (5hmC), recruits PRMT1 as part of the methylosome complex, which then stimulates the expression of cancer-related genes EGFR, AKT3, cyclin-dependent kinase (CDK6), cyclin D2 (CCND2), and rapidly accelerated fibrosarcoma oncogene B-homolog (BRAF), through the methylation of H4R3. Knock-down of CHTOP decreases spheroid formation of GBM cells (GB2). The production of 5hmC from 5-methylcytosine (5mC) is dependent upon the ten-eleven translocation (TET) family of enzymes. The activity of the TET enzymes is inhibited by 2-hydroxyglutarate, a product of the mutated version of the IDH1 enzyme, a major prognostic marker of GBM. The involvement of PRMT1 could provide an additional mechanism for the positive prognosis seen in GBM patients bearing a mutant IDH. This idea is supported by previous findings of interactions between PRMT1 and CHTOP in chromatin (van Dijk et al. 2010; Izumikawa et al. 2014).

Co-immunoprecipitation, western blot, silver staining and mass spectrometry have been used to identify possible binding partners of PRMT1 in glioma cells (Wang et al. 2012b). In that study, Sec23 homolog 23-interacting protein (SEC23-IP), ankyrin repeat and KH domain containing 1-eukaryotic translation initiation factor 4E-binding protein-3 (ANKHD1-EIF4EBP3) protein, and 1-phosphatidylinositol-3-phosphate 5-kinase were found to have at least two methylated arginine sites in U-87MG cells.

Altogether, the current literature suggests that PRMT1 plays a role in GBM pathogenesis. This is based on the observations of increased PRMT1 expression in GBM cells and tissue, GBM cells dependency on PRMT1 activity for proliferation, and the correlation of PRMT1 expression with disease stage and poor patient survival. A number of interacting partners and targets that contribute to its activity in GBM have also been identified, perhaps the most interesting being CHTOP and 5hmC, the activity of which may be altered in mutant IDH1 GBM.

### PRMT2 in GBM

PRMT2 was first discovered in 1997 (Katsanis et al. 1997). It is mainly localised to the nucleus but is also found at lowered levels in the cytoplasm (Kzhyshkowska et al. 2001). PRMT2 contains a unique Src homology 3 (SH3) domain that enables binding to proline rich motifs on other proteins (Pawson and Gish 1992; Cura et al. 2017). Despite having high sequence homology with the other enzymes, PRMT2 was initially not thought to possess methyltransferase activity (Scott et al. 1998). Later studies revealed a weak methyltransferase activity of PRMT2 towards histones (Lakowski and Frankel 2009; Blythe et al. 2010; Su et al. 2014).

In a study carried out by Dong et al. PRMT1, -2, -4 and -6 mRNA expression were found to be correlated with tumour grade and high expression was determined to be predictive of patient prognosis (Dong et al. 2018). On the other hand, expression of PRMT5, -7, -8 and -9 correlated with a more favourable prognosis. Dong et al. found that knocking down PRMT2 by shRNA resulted in loss of proliferation in both T98G and U-87MG cells, each having different phosphatase and tensin homolog (PTEN) status (wild type and deleted, respectively). Quantification of PRMT2 in 21 cases of glioma of different grades by immunohistochemistry showed increased PRMT2 expression in higher grade samples. Knock-down of PRMT2 by shRNA in T98G and U-87MG cells led to a decrease in cell number and a decrease in spheroid formation, suggesting a role in self-renewal. To further investigate this, limiting dilution assays were performed and it was shown that cells depleted of PRMT2 were less capable of producing spheroids and also indicated decreased expression of stem cell-associated genes, as judged by quantitative reverse transcription PCR. Oncogenic transcriptional programmes were also reduced including PI3K-AKT, MAPK, Janus kinase (JAK)-STAT and Wnt signalling pathways. T98G and U-87MG cells were transduced with a luciferase expressing virus with either shScrambled or shPRMT and injected into mice. Cells depleted of PRMT2 were less able to form tumours and the mice displayed significantly prolonged survival. These changes were linked with the methylation mark on H3R8me2a (Dong et al. 2018). Although there are limited studies investigating PRMT2 in GBM, it is clear that PRMT2 contributes to the pathogenesis of GBM, most likely through promoting cell stemness.

### PRMT5 in GBM

PRMT5 is the major methyltransferase responsible for SDMA within the cell. It can be found expressed in both the nucleus (Lacroix et al. 2008) and cytoplasm, including within the Golgi apparatus (Zhou et al. 2010), and has a role in a number of processes. PRMT5 is perhaps the most studied of the PRMTs, with roles found in a great range of cellular functions including transcription, translation, splicing, DNA damage repair and growth factor signalling. PRMT5 has been shown to be expressed and active in normal neuronal cells and has a role in neuronal stem cell proliferation (Han et al. 2014; Chittka 2010; Chittka et al. 2012). When dysregulated, PRMT5 has the potential to cause uncontrolled cell growth in neuronal cells.

PRMT5 has been found to be overexpressed in numerous GBM cell lines when compared to those originating from normal brain tissue (Yan et al. 2014a). More importantly, a significant increase in expression of PRMT5 was observed in grade III and IV astrocytoma patients when compared with normal or grade I and II brain tissue by immunohistochemistry. PRMT5 mRNA was not found to be differentially expressed, however, in a later study, PRMT5 mRNA expression was found to have a more favourable patient survival (Dong et al. 2018). Dysregulation of protein expression may occur at the level of translation, most likely through one of the regulatory mechanisms described in “Alternative splicing”, “Micro RNAs”, “Post-translational modifications of PRMTs”, “Cross-talk between ArgMe and other PTMs”, and PRMT–protein interactions sections above (Han et al. 2014).

Braun et al. observed a loss of proliferation in U-87MG cells following treatment with the PRMT5-specific inhibitor EPZ015666, indicating a dependency on PRMT5 activity (Braun et al. 2017). Cell cycle profiling suggested this reduction in growth was due to activation of senescence. The upregulation of senescence-associated markers (beta-galactosidase positive cells) was reported, although this was not accompanied by an increase in G2/M cells which would otherwise indicate activated p53 and apoptosis.

Due to its role in neuronal stem cell proliferation and the increasing significance of cancer stem cells, Banasavadi-Siddegowda et al. investigated the differential effects of PRMT5 silencing through pooled siRNA in stem-like and differentiated glioblastoma cells (Banasavadi-Siddegowda et al. 2017). Loss of PRMT5 caused a reduction in cell proliferation in stem-like cells only but not through an increase in apoptosis. Following differentiation of the cells by prolonged incubation with serum-containing media, sensitivity to PRMT5 knock-down was achieved. Cell cycle analysis by propidium iodide staining revealed a G1/S cell cycle arrest in only stem-like cells suggesting that the apoptosis in differentiated GBM cells was cell cycle independent. An increase in senescence using the beta-galactosidase assay was observed in stem-like GBM cells. This was supported by cell cycle arrest, decreased proliferation and increased cell size. Further investigation showed an elevation in Akt activation and its upstream target PTEN following knock-down of PRMT5 in stem-like GBM cells. This was in contrast to the results from Han et al., where no increase in Akt activation following PRMT knock-down by shRNA had been observed in U373MG cells (Han et al. 2014). Proliferation of these cells was rescued following a combinational knock-down of both PRMT5 and PTEN, indicating a role for PTEN in the induction of senescence in the absence of inhibition by PRMT5.

Han et al. showed an increase in ERK1/2 signalling following knock-down of PRMT5 through shRNA (Han et al. 2014). As over-activation of this pathway has previously been shown to cause cell death in GBM cells, Han et al. proposed that this mechanism may be regulated by PRMT5 to allow for tumour growth. More indirect evidence of the relevance of PRMT5 in GBM includes the report that the long non-coding RNA, small nucleolar RNA host gene (SNHG16), has been found to be upregulated in glioma tissues, such as GBM. SNHG16 was shown to have oncogenic properties by “sponging” cellular miR-4518, a known regulator of PRMT5 expression (Lu et al. 2018).

Work by Mongiardi et al. has suggested a role for Myc in the tumorigenesis nature of PRMT5 in GBM (Mongiardi et al. 2015). Expression of the Omomyc protein, an effective inhibitor of specific N-Myc interactions, correlated with a decreased prevalence of the H4R3me2 histone mark, a PTM dependent upon PRMT5, in both U-87MG and patient-derived cells. Inhibition of Myc, as expected, resulted in a loss of expression of its target proteins, carbamoyl-phosphate synthetase 2, aspartate transcarbamylase and dihydroorotase (CAD) and cyclin D1. Their expression was recovered, however, through the knock-down of the PRMT5-associated protein CORP50. These findings are supported by the known stabilising interactions between Myc and PRMT5 (Park et al. 2015). Further investigations suggested a role for PRMT1 in these interactions, and it was found that Myc was both symmetrically (by PRMT5) and asymmetrically (by PRMT1) dimethylated in GBM stem cells and that these modifications were required for Myc activity and turnover, respectively (Favia et al. 2019). PRMT5 has also been associated with Myc-driven primary medulloblastoma tumours (Chaturvedi et al. 2019). This group has shown that PRMT5 is overexpressed in these tumours, compared to normal tissue, and that PRMT5 expression inversely correlated with survival. Knocking down PRMT5 in Myc-driven medulloblastoma cells led to a significant inhibition of cell growth (Chaturvedi et al. 2019).

Finally, a recent paper by Holmes and colleagues explored possible synergies between PRMT5 inhibition and other treatments in glioblastoma (Holmes et al. 2019). This is an important issue, because it is unlikely that any novel treatment will be introduced to neurooncology clinics unless it has been investigated in combination with the standard of care. Although combinations of PRMT inhibitors and TMZ have not yet been fully investigated, the paper by Holmes et al. sheds light on a different class of inhibitors that has also been explored for GBM treatment through clinical trials, that is, mammalian target of rapamycin (mTOR) inhibitors. They found that PRMT5 activity was stimulated by mTOR inhibitors, which could explain resistance to mTOR inhibition, and that the concurrent inhibition of PRMT5 and mTOR pathways led to synergistic anti-proliferative effects both in GBM cell lines and in a xenograft model (Holmes et al. 2019).

To summarise, PRMT5 is expressed at greater levels in both GBM cell lines and patient tissue and PRMT5 expression correlates with disease stage. Similar to PRMT2, PRMT5 activity in GBM cells has been linked to cell stemness and tumour cell ability to self-renew. A number of mechanisms have been suggested for this activity including through the regulation of PTEN and Akt signalling as well as Myc and ERK1/2 signalling.

### PRMT8 in GBM

PRMT8 is a type I enzyme with a similar structure to PRMT1, sharing greater than 80% sequence homology and differing only by its N-terminal domain. Unlike PRMT1, however, PRMT8 is exclusively expressed in CNS tissue (Lee et al. 2005). PRMT8 also differs in that it is membrane bound due to its myristoylation motif found on its N-terminal domain. In contrast to most other PRMTs, PRMT8 seems to have a reduced transcript expression in GBM patient tissue when compared with normal tissue, suggesting it may be down regulated during tumour development (Simandi et al. 2015). This decrease in expression was accompanied by a significant increase in the expression of C-X-C chemokine receptor type 4 (Cxcr4) and epidermal growth factor-containing fibulin-like extracellular matrix protein 1 (Efemp1). Silencing of PRMT8 in embryonic stem cell-derived neurons, through the use of shRNA, caused a differential expression pattern in genes including Cxcr4 and Efemp1, which are known to play a role in glioma (Idbaih et al. 2008). PRMT8 mRNA expression was found to confer a favourable patient survival in GBM patients (Dong et al. 2018).

Knock-down of a previously unknown PRMT8 transcript variant, named PRMT8 variant 2, was shown to reduce proliferation of the GBM cell line U-87MG (Hernandez and Dominko 2016). This variant of PRMT8 shows nuclear localisation, due to the loss of the myristoylation motif in the N-terminal domain, and most likely acts through epigenetic regulation of gene expression (Hernandez et al. 2017). A similar loss in proliferation, however, was also observed in non-tumorigenic cells.

In conclusion, PRMT8 does not seem to play a role in the pathogenesis of GBM and is, in fact, associated with a better patient prognosis, although the significance of this is still unclear. However, upon nuclear localisation through the loss of its myristoylation motif, PRMT8 appears to have a switch in molecular targets, which confers a tumorigenic phenotype, most likely similar to PRMT1.

## Conclusion and perspective

PRMTs present a novel target for GBM treatment due to their ability to augment a vast variety of cellular processes related to cell growth, including growth factor signalling, DNA damage repair and proliferation, coupled with their increased expression in GBM tumour cells and tissues. GBM cells may well, therefore, be dependent on PRMT expression and function and, consequently, PRMT inhibition results in cell death or reduced proliferation (Wang et al. 2012c; Dong et al. 2018; Braun et al. 2017). Tools to investigate ArgMe have been developed over the past decade and include PRMT inhibitors (see sections “PRMT inhibitors” and “Clinical trials involving PRMT inhibitors”). Recent examples exist of projects that (1) have used technologies for enrichment in peptides bearing ArgMe (Musiani et al. 2019; Onwuli et al. 2019), (2) have generated enzymatic dead PRMT versions (Radzisheuskaya et al. 2019), (3) have created mutant, methylation-deficient proteins (Jeong et al. 2019; Liu et al. 2020b), (4) have used PRMT KO mice (Cheng et al. 2020), (5) have generated antibodies specific against ArgMe sites (Kumar et al. 2020) and (6) have developed kinetic assays for measuring PRMT activity (Hevel and Price 2020). This list is not comprehensive and aims to provide the reader with research ideas that have recently been used, successfully.

However, a limitation of current investigations of ArgMe in GBM is the common use of classic, well-established cell lines and culture models, which lack tumour microenvironment and patient specificity. From a research perspective, recent advances in biomicrofluidics have developed ever-improving organ-on-chip models to investigate patient-specific GBM biopsies (Olubajo et al. 2020). This could be adapted by multidisciplinary teams including the neurooncology and biochemical communities to deliver greater understanding, at the molecular and cellular level, of PRMT interactions, activity and relevance in GBM cells and patient samples. Models to investigate the permeability of PRMT inhibitors through the blood brain barrier are also needed.

Developing recent research efforts (Onwuli et al. 2017) towards the solid identification of the brain arginine methylome in health and disease can also provide clear and specific targets, either at the protein and ArgMe site level or at the level of protein networks and pathways. In this sense, although there has been considerable success in the past few years towards the development of synthetic PRMT inhibitors, these have been directed towards specific PRMTs and very few have targeted PRMT–protein interactions and other regulatory pathways of PRMT activity, such as those reviewed in this publication. This opens research opportunities to increase the pharmacological repertoire towards modulation of ArgMe. Specificity is key and the development of inhibitors against not only specific PRMTs but also specific ArgMe events would greatly advance the field, including inhibitors that target cross-talk events between ArgMe and other PTMs. On the note of specificity, the introduction of targeted therapies that can be specifically directed against brain tumour cells, and therefore, able to cross the blood brain barrier, is another such research opportunity requiring a multidisciplinary team of chemists, biochemists and cancer biologists. As highlighted above, new therapies against GBM will most likely be introduced alongside TMZ. Therefore, it is important that research efforts investigate the combination of novel possible therapies, such as PRMT inhibitors, with standard of care interventions, such as TMZ and radiation.

From a clinical perspective, ongoing clinical trials will help investigate the efficacy of PRMT inhibitors in patients with GBM. A key question is: which patients are likely to benefit from PRMT inhibitors? Genetic biomarkers that predict patients that respond to TMZ are available and include levels of O^6^-methylguanine-DNA methyltransferase (MGMT) promoter methylation. Recently, the possibility that expression levels of the methylthioadenosine phosphorylase gene MTAP may be a possible biomarker for patient selection for treatment with PRMT inhibitors has been put forward (Fedoriw et al. 2019). This pharma-led investigation found an increased sensitivity of brain tumour cells towards PRMT inhibitors upon deletion of MTAP, presumably through accumulation of 2-methylthioadenosine, a PRMT5 endogenous inhibitor (Fedoriw et al. 2019). Consistent with this, subsequent clinical trials have included MTAP deficiency as a patient selection biomarker (NCT03666988, Table 2). The presence of p53 mutations has also been proposed as a predictor of sensitivity to PRMT5 inhibition, based on results using a wide panel of cancer cell lines (Gerhart et al. 2018). Given that PRMT expression tends to correlate with GBM stage and clinical outcome (see “Arginine methylation in GBM” section), biopsy PRMT levels can be biomarkers for patient selection for PRMT inhibitor treatment, and for prognosis. Finally, as discussed in “PRMT1 in GBM” section, high levels of CHTOP and 5hmC are thought to be drivers of glioblastomagenesis through PRMT1-mediated gene activation (Takai et al. 2014) and, as such, may be useful predictors of the response of patients to PRMT inhibitors.

The involvement of several pharmaceutical companies and research institutions holds the promise of translation of PRMT inhibitors from bench to bedside. However, important questions remain not only at the molecular and mechanistic levels, but also from a clinical perspective. For example, how is target inhibition in GBM trial samples and then clinical samples to be assessed? A possibility would be to test ArgMe levels and PRMT activity directly in resected biopsies, for instance using antibodies against ArgMe and PRMT assays, respectively. The levels of methylation of specific proteins well known to be methylated, such as RNA binding proteins, could also serve as useful biomarkers. A more intriguing possibility, at least conceptually, would be to measure methylarginine metabolites (mMA, aDMA, sDMA) locally, or in circulation. PRMT inhibitors are normally administered systemically (either intravenously or orally) and, therefore, whole-organism PRMT activity levels are likely to decrease. Because mMA, aDMA and sDMA are the proteolytic products of methylated proteins, their local and plasma concentrations may decrease following systemic and prolonged PRMT inhibition. Would lower methylarginine concentrations correlate with better clinical outcome? These and other questions warrant further investigations on the role of ArgMe, methylarginine metabolites and PRMT inhibition in brain tumours. Overall, we believe that the combination of research approaches and clinical trials will help dissect the effect of PRMT inhibitors on GBM and their potential for translation into the clinics as personalised ‘silver bullet’ treatments (Dilworth and Barsyte-Lovejoy 2019) that some GBM patients can hopefully benefit from.
